# Expanding the Horizons of CAR-T Cell Therapy: A Review of Therapeutic Targets Across Diverse Diseases

**DOI:** 10.3390/ph18020156

**Published:** 2025-01-24

**Authors:** Alejandrina Hernández-López, Alberto Olaya-Vargas, Juan Carlos Bustamante-Ogando, Angélica Meneses-Acosta

**Affiliations:** 1 Laboratorio 7 of Biotecnología Farmacéutica, Facultad de Farmacia, Universidad Autónoma del Estado de Morelos, Cuernavaca 62210, Mexico; alejandrina.hernandez@uaem.edu.mx; 2 Consejo Nacional de Humanidades, Ciencias y Tecnologías (CONAHCYT), Mexico City 03940, Mexico; 3 Programa de Trasplante de Células Madre Hematopoyéticas y Terapia Celular, Instituto Nacional de Pediatría, Mexico City 04530, Mexico; alberto.olaya@yahoo.com.mx; 4 Laboratorio de Investigación en Inmunodeficiencias y Departamento de Inmunología Clínica, Instituto Nacional de Pediatría, Mexico City 04530, Mexico; drbustamante_inp@hotmail.com

**Keywords:** Chimeric Antigen Receptor, new CAR targets, CAR-T cell therapy

## Abstract

CAR-T cell therapy has shown promising results in treating malignant hematologic diseases. The principle of this therapy is based on the use of genetically modified T lymphocytes to express a Chimeric Antigen Receptor (CAR) on their membrane that specifically recognizes an antigen predominantly expressed on target cells. The molecular design of the CAR, along with advancements in molecular techniques and the development of “omics”, has opened the possibility of discovering new therapeutic targets and thereby expanding the range of diseases treated with CAR-T cells beyond the use of anti-CD19 and anti-BCMA for hematologic cancer. This review summarizes the novel therapeutic targets that are currently used in clinical trials with CAR-T cell therapy on autoimmune diseases and other challenging conditions, such as cardiac fibrosis, and different infections. Additionally, challenges and novel opportunities are discussed for expanding clinical access to this innovative therapy.

## 1. Introduction

Currently, biotechnological advancements in molecular techniques, the development of “omics”, and cell culture technology for supporting health sciences have evolved toward personalized medicine, paving the way for the use of genes, cells, or tissues as treatment strategies. These types of therapies have been nominated as ’Advanced Therapies’ and represent innovative strategies that offer new opportunities for survival with promising results in patients with genetic, malignant, chronic-degenerative, and infectious diseases. In fact, advanced therapy medicinal products (ATMPs) have demonstrated significant potential as therapies where conventional treatment alternatives are not effective. Some of the most promising ATMPs are Chimeric Antigen Receptor-T cells (CAR-T), which constitute a novel systemic therapy in which T cells are engineered to express a Chimeric Antigen Receptor (CAR) that targets the membrane antigens present in damaged cells. The chimeric antigen receptor (CAR) is a synthetic receptor designed to redirect the specificity and functionality of T lymphocytes and other immune cells to recognize specific ligands [1]. The first CAR-T cell therapy targeting CD19+ B-cell hematological malignancies has demonstrated significant success in clinical applications, highlighting its potential as an effective therapeutic strategy. This success opens numerous avenues for targeting additional antigens; however, several important challenges at various levels must be addressed. Since 2017, when CAR-T cells were first approved as a treatment for leukemia and lymphoma, several clinical trials have been ongoing to evaluate the use of such therapies for the treatment of different hematologic and solid malignancies, demonstrating the potential of this therapy in the field of oncology [2]. Nowadays, in other areas beyond hematological malignancies, such as solid tumors, autoimmune diseases, and infectious disorders, other types of CARs are expressed in novel types of cells, including natural killer (NK) cells, macrophages, regulatory T cells (Tregs), and γ δ T cells that are used in clinical research [3]. Novel research for treating a variety of diseases using different types of cells promises a very important increase in their use even though important challenges have to be solved such as cellular specificity, safety and efficacy of the product, and, in a wider sight, cost and worldwide coverage.

## 2. CAR Molecule Design

**Figure 1:**
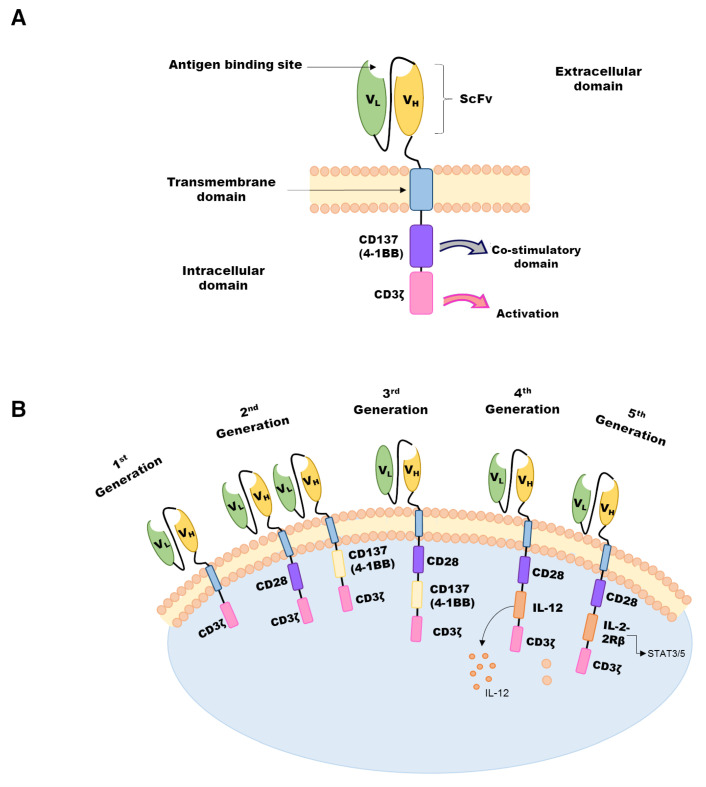
Structure and Evolution of Chimeric Antigen Receptor (CAR) Design. (**A**), The CAR is composed of an extracellular, transmembrane, and intracellular domain. The extracellular domain consists of heavy and light chains of an immunoglobulin with the ability to specifically bind to a target molecule expressed on the surface of tumor cells, and the intracellular domain drives the activation and amplification of CAR-T cells. (**B**), Evolution in CAR design with modifications from the first to the 5th generation. V_L_, Light variable chain; V_H_, Heavy variable chain; ScFv, Single-chain variable fragment; IL, Interleukin.

Considering the potential action of different expressed CARs in different types of cells, the design of the CAR structure is crucial, and it is of vital importance as each component contributes to its effector function, efficacy, and toxicity [4]. The prototypical molecular CAR design consists of three different regions: (1) an extracellular antigen-binding domain, (2) a transmembrane segment, and (3) an intracellular signaling domain capable of activating the lymphocyte as soon as the antigen is detected [5]. The extracellular antigen-binding domain is composed of a monoclonal antibody variable heavy (VH) and variable light (VL) chains, forming a single-chain variable fragment (scFv), which is a binding site to interact with the target antigen [6] (Figure 1A). This ectodomain is linked by a hinge or spacer region to the transmembrane domain and an intracellular signaling domain [7]. The appropriate hinge domain design can impact the capacity to enable recognition of target antigens that are otherwise sterically inaccessible, providing flexibility and influencing CAR-T efficacy [4,8]. The intracellular signaling/activation domain plays a crucial role in transmitting signals to activate the effector function of CAR-T cells [9]. Usually, CAR intracellular domains are derived from co-stimulatory molecules from the CD28 family (including CD28 and ICOS) or the tumor necrosis factor receptor (TNFR) family of genes (including 4-1BB, OX40, or CD27) [10]. This composition has been optimized by integrating additional co-stimulatory signals in tandem to achieve clinically effective activation and persistence, thus leading to the development of more effective and safer CARs, which are classified from the first to the fifth generations (Figure 1B) [11].

## 3. Evolution of CAR Structure

The first CAR was developed between 1989 and 1993, when Eshhar and colleagues combined the cytotoxic potential of a T cell with the specificity of an antibody [5,12], adding a cytoplasmic domain containing a CD3ζ (signaling domain but without extra co-stimulatory molecules) [13]. However, this scientific development did not achieve clinical impact because effective T-cell responses could not be generated by signaling from CD3ζ alone, resulting in minimal or no clinical efficacy [14,15]. Following the proof-of-concept, with the results obtained with first-generation Chimeric Antigen Receptors (CARs), researchers sought to enhance their efficacy and effectiveness. Drs. Rosenberg, June, Sadelain, Irvin, and Weiss demonstrated that T-cell activation independent of the TCR was achievable by linking the extracellular domains to the cytoplasmic domain of the CD3ζ chain and incorporating a co-stimulatory region (CD28 or 4-1BB). This approach led to the development of the second generation of CARs [16]. In 2011, Savoldo and col. [17] highlighted the significance of co-stimulatory domains by employing a combination of first- and second-generation CAR-T cells for the treatment of lymphoma patients, which improved the persistence of CAR-T cells. These second-generation CARs conferred a proliferative advantage over their first-generation counterparts and resulted in a higher release of immune-stimulating cytokines while maintaining their cytolytic potential [16]. Subsequently, the third generation of CARs emerged, building upon the designs of second-generation CARs by incorporating both CD28 and 4-1BB co-stimulatory domains. It is noteworthy that the fourth and fifth generations of CARs are also based on second-generation designs. Fourth-generation CARs, often referred to as TRUCKs (T cells redirected for universal cytokine-mediated killing), are engineered to promote the release of interleukin 12 (IL-12) as part of the signaling cascade triggered by CAR activation. This design aims to attract additional immune cells, such as natural killer (NK) cells, and remodel the tumor microenvironment to enhance therapeutic effects [11,18,19]. Fifth-generation CARs incorporate an additional intracellular domain compared to previous generations, this domain is called the IL-2Rβ (human IL-2 receptor β chain), and it is added with a binding site for the STAT3/5 transcription factor [11]. This design enables the production of memory T cells, reactivating and stimulating the immune system.

## 4. CAR-T Cells’ Mechanism of Action

The CAR insertion into T lymphocytes endows them with major efficacy in combating cancer and specificity to recognize the target antigen independently of Major Histocompatibility Complex (MHC) antigen presentation. After infusion, CAR-T cells can persist in the bloodstream for months, leading to long-term remissions in certain types of hematologic cancers [20].

Prior to the infusion, patients typically undergo a round of chemotherapy to deplete other immune cells, which facilitates the proliferation of CAR-T cells. Once reintroduced into the patient’s bloodstream, CAR-T cells travel throughout the body, homing in on the target antigen expressed on the surface of cancer cells. The design of the CAR is tailored to recognize a specific antigen, chosen based on the type of cancer being treated. This specificity enhances the ability of CAR-T cells to detect and eliminate malignant cells effectively.

Upon recognition of the antigen, the CAR-T cells are activated via intracellular signaling domains that include a portion of the T-cell receptor (TCR) known as the CD3 zeta chain (3ζ). This component contains three immunoreceptor tyrosine-based activation motifs (ITAMs), which can initiate a robust signaling cascade even in the absence of the complete TCR-CD3 complex (which includes the γ, δ, and ε chains). The signaling process begins with the phosphorylation of ITAMs by the protein tyrosine kinase (Lck) [21]. Following this initial activation, co-stimulatory domains provide a second signal essential for the complete activation of CAR-T cells. This signaling cascade is crucial for their proliferation, evasion of anergy, and apoptosis, as well as for producing interleukin-2 (IL-2) [22]. Additionally, it triggers the cytotoxic potential of CAR-T cells against the malignant cell. Once fully activated, CAR-T cells release large quantities of soluble factors, including perforin, granzymes, and various cytokines. This release leads to tumor pyroptosis characterized by the release of inflammatory molecules [22,23]. Concurrently, these proteins initiate a cytokine cascade that attracts other immune cells to the tumor site, further promoting tumor elimination. While this cascade is instrumental in the successful eradication of malignant cells, it also induces significant systemic inflammation, which is responsible for many of the adverse effects associated with CAR-T cells [23,24].

## 5. Challenges with Targeting T Cells with CAR-T Cell Therapy

Most commercial CAR-T cell therapies target B cells. However, targeting T cells may be beneficial in T cell-derived malignancies and autoimmune or inflammatory diseases. Directing CAR-T cells against pathogenic T cells poses unique challenges due to the shared expression of surface antigens between effector and targeted cells. The associated clinical problems are (a) fratricide, where CAR-T cells may recognize and destroy each other if the target antigen is also expressed on the surface of the CAR-T cells. For example, therapies targeting CD7 or CD5 in T-cell malignancies have encountered fratricide due to the expression of these antigens on both malignant and non-malignant T cells, including CAR-T cells [25,26]. (b) Immunosuppression, caused by T-cell malignancies and autoimmune disease microenvironments, can inhibit CAR-T cell expansion, persistence, and function mediated by the production of suppressive cytokines (e.g., TGF-β, IL-10) and the recruitment of regulatory T cells or myeloid-derived suppressor cells [27]. (c) Antigen escape, caused by the dynamic expression of antigens on malignant T cells, reducing the efficacy of CAR-T therapies. This problem is particularly relevant for T-cell lymphomas and other proliferative diseases with high mutation rates [28].

## 6. CAR-T Cells as a Treatment for Hematologic Cancer

Currently, CAR-T cells are employed as a second or third line of treatment, primarily when cancer cells evade detection by the immune system. This immune escape occurs because immune cells fail to recognize tumor cells effectively. As a result, these cells are unable to proliferate for attacking and eliminating cancer cells or when there are no other therapeutic options [29,30]. The first clinical application of CAR-T cells was made possible by the specialized team at the University of Pennsylvania and the Children’s Hospital of Philadelphia, where Carl June and David Porter led the administration of second-generation CAR-T cells to adult patients with chronic lymphocytic leukemia (CLL) in 2011 and, together with Stephan Grupp, in 2012, to pediatric patients with acute lymphoblastic leukemia (ALL) [5]. The results revolutionized the treatment of relapsed or refractory malignant hematological neoplasms. Although the first clinical CAR-T cell use occurred over a decade ago, it was not until 2017 that the FDA and EMA approved the use of tisagenlecleucel (Kymriah) for the treatment of pediatric and young adult patients with relapsed or refractory acute lymphoblastic leukemia, thus opening the field for the use of CAR-T cells. During the past decade, CAR-T cell therapy has achieved good results in the treatment of hematological tumors, leading to the approval by the FDA and EMA of, up to 2024, six CAR-T cell-based therapies for the treatment of acute lymphoblastic leukemia, chronic lymphocytic leukemia, lymphoma, and, more recently, multiple myeloma [30,31,32] (Table 1).

CAR-T cell therapy in acute myeloid leukemia (AML) remains challenging due to the heterogeneity in target antigen expression across leukemic cells and patients, the difficulty of controlling on-/off-target tumor effects, and the immunosuppressive tumor microenvironment. Damiani and Tiribelli (2024) discuss the current state of CAR-T cell therapy for AML, highlighting its potential, limitations, and new strategies to enhance its effectiveness [33]. One promising approach, led by Nirali N. Shah at the Pediatric Oncology Branch, involves a clinical trial of CAR-T cell therapy for relapsed or refractory AML patients post-allogeneic hematopoietic stem cell transplant (NCT05984199).

## 7. Emerging and Potential Applications of CAR-T Cell Therapy

Although the use of CAR-T cells has yielded promising outcomes in hematological malignancies, there are potential challenges and significant limitations that persist, particularly in the treatment of solid malignancies. These limitations include heterogeneous antigens, the immunosuppressive tumor microenvironment, associated risks with on-target/off-tumor effects, the infiltration of CAR-T cells, immunosuppressive checkpoint molecules, and cytokines [34]. To overcome these challenges, many strategies are being explored, such as the expression of CARs in other immune cell types; the use of protein kinase A inhibition; gene-editing methods to knockout PD-L1; and novel CAR designs, such as multi-target CAR-Ts or dual-target combinations, which can broaden antigen coverage and improve CAR-T efficacy, persistence, and specificity, thereby avoiding tumor heterogeneity [35]. Additionally, the use of oncolytic viruses such as adenovirus, vaccinia virus, and lentivirus has been proposed to enhance CAR-T cell trafficking and infiltration into tumor cells [9,36]. To date, the field of CAR-T cell therapy is experiencing remarkable progress, with ongoing research and clinical investigations driving significant advancements. The search for new therapeutic targets to expand the applications of CAR-T cells continues to advance at a remarkable pace. Researchers worldwide are actively identifying specific antigens linked to a diverse range of diseases, including autoimmune disorders and chronic infections. This ongoing effort highlights the immense potential of CAR-T cell therapy to revolutionize the treatment of an ever-expanding spectrum of conditions while enhancing its efficacy and safety. Table 2 summarizes registered clinical trials exploring the use of novel therapeutic targets beyond hematological cancers (Table 2).

## 8. Breaking New Ground

Currently, CAR-T cell therapy is being investigated for the treatment of autoimmune, cardiac, and infectious diseases, including viral infections such as those caused by human immunodeficiency virus (HIV), hepatitis B and C viruses, human cytomegalovirus, and even opportunistic infections such as tuberculosis and aspergillosis [31,37]. Therefore, CAR-T cell therapy promises continuing growth and the development of treatments for human diseases that were previously incurable.

### 8.1. Autoimmune Diseases

The field of autoimmune diseases has made significant strides, driven by advancements in CAR-T cell therapy. Systemic autoimmune diseases are characterized by the production of autoantibodies by plasma B cells and autoreactive T lymphocytes, which target and damage the body’s tissues [38]. In many cases, the progression of autoimmune diseases is heavily dependent on the continued production of autoantibodies by B cells. Consequently, CAR-T cell therapy aimed at specific or broad B-cell depletion offers a promising avenue for treating refractory autoimmune diseases. This approach not only addresses immune dysregulation but also represents a potentially curative strategy for these challenging conditions [3]. A specific example of its potential use is the molecular mechanism by which CAR-T cells contribute to restoring the immune response using CD19+. This is as follows: (1) Specific antigen recognition with a CAR receptor, CAR receptor is specific for an antigen expressed in self-reactive immune cells, such as CD19 in B cells. In the case of diseases such as lupus or rheumatoid arthritis, where B cells produce autoantibodies that damage tissues, CAR-T cells directed against CD19 eliminate these pathogenic B cells; (2) stimulation of T Cell activation signaling, once the CAR-T cells attach to their target (e.g., CD19 in B cells), the CAR receptor activates a cascade of intracellular signaling in the T cells that results in their activation and proliferation. CAR-induced signaling usually involves intracellular domains of co-stimulating molecules, such as CD28 or 4-1BB, and activation signals (such as CD3ζ). This ensures a powerful and sustained activation of CAR-T cells to eliminate self-reactive cells; (3) Depletion of autoreactive cells, the activation of CAR-T cells triggers cytotoxic mechanisms to destroy cells that express the target antigen, including the release of granzymes and perforins, proteins that induce apoptosis (programmed cell death). By eliminating self-reactive B cells, the immune system is “reprogrammed” and stops producing autoantibodies that attack healthy tissues, which reduces inflammation and symptoms of autoimmune disease; (4) Induction of immune remission, the elimination of self-active B cells also allows the restoration of a population of regulatory and tolerant B cells when the immune system recovers. This immune “restart” helps to establish a longer remission of the disease. In some studies, it has been observed that the patient’s immune system adapts after CAR-T therapy and can recover its normal function without generating autoantibodies; (5) Expansion and persistence of CAR-T cells, the ability of CAR-T cells to expand and persist in the patient’s body is critical to maintaining their long-term effectiveness. Some CAR designs include co-stimulation domains, such as 4-1BB, which improve the persistence and durability of the immune response without causing the rapid exhaustion of T cells [3,4,18].

CAR-T cell therapy is emerging as a promising treatment option for various autoimmune diseases, with several clinical trials currently underway. Notable progress has been made in systemic *lupus erythematosus* (SLE), where anti-CD19 CAR-T cells have demonstrated potential to eliminate autoreactive B cells and induce sustained remission in refractory cases [39]. Similarly, in *rheumatoid arthritis* (RA), *myasthenia gravis* (MG), neuromyelitis optical spectrum disorders (NMOSDs), idiopathic inflammatory myositis, and Crohn’s disease, CAR-T therapies targeting CD19+, CD7, and CD20+ B cells are being explored to reduce autoantibody production and alleviate disease symptoms [40]. Clinical trials are also investigating CAR-T cells in multiple sclerosis, focusing on modulating autoreactive T cells to prevent demyelination in the central nervous system [41]. Recent advancements focus on using chimeric autoantibody receptor T (CAAR-T) cells and CAR-T regulatory T cells (CAR-Tregs) to restore immune tolerance in conditions like *pemphigus vulgaris (PV),* a rare skin-blistering disease caused by autoantibodies against desmoglein 1 and 3 (DSG1/3) [42,43]; or to combat muscle-specific tyrosine kinase *myasthenia gravis* (MuSK-MG), a life-threatening muscle disorder characterized by muscle weakness due to anti-MuSK autoantibodies [43,44,45] (Table 2).

Given the remarkable potential demonstrated by CAR-T cell therapy, numerous preclinical studies are actively investigating novel therapeutic targets for autoimmune diseases. These studies aim to expand the applications of CAR-T cells by focusing on specific immune components implicated in various autoimmune conditions. Preclinical research continues to be focused on antigens associated with systemic *lupus erythematosus* for depleting B cells and reducing autoantibody production. In *myasthenia gravis*, CD137-based CAAR-T cell therapy has achieved antigen-specific B-cell depletion. For pemphigus vulgaris, DSG3 CAAR-T cells effectively inhibited antibody responses [3]. In type 1 *diabetes mellitus* (T1DM), targeting the I-Ag7-B:9–23 complex or clonotypic T-cell receptors (TCRs) has demonstrated the ability to delay disease onset in murine models. Furthermore, redirecting CAR regulatory T cells (CAR-Tregs) toward pancreatic β-cells has shown potential to prevent diabetes progression in diabetic mouse models [46]. These findings emphasize the versatility of CAR-T cell therapy as a promising approach for addressing a range of autoimmune diseases.

These ongoing studies underscore the versatility of CAR-T technology and its potential to revolutionize the management of autoimmune diseases. Preclinical and early clinical data show promise, with some patients achieving drug-free remission after CAR-T cell therapy, although more research is needed to evaluate long-term safety and efficacy [47]. There are many challenges to address before CAR-T therapies become widely applicable for autoimmune diseases, including that the CAR-T cell therapy must be precisely designed to target only the specific cells responsible for disease without disrupting the rest of the immune system. Low specificity or regulation could lead to severe side effects, such as opportunistic infections (from excessive immune suppression), cytokine release syndrome, and improved stability of CAR-T cells to ensure sustained therapeutic benefits [48]. Another significant challenge is the immunogenicity of CAR-T cells, due to the induction of humoral and/or cellular immune responses against components of the CAR construct that may be perceived as foreign. This immune activation could potentially exacerbate autoimmune diseases [49]. Consequently, developing CAR-T therapies for autoimmune diseases requires carefully balancing therapeutic efficacy and risk mitigation. Approaches such as designing regulable CAR-T cells or targeting highly specific antigens are being actively explored to address these challenges.

### 8.2. Cardiac Fibrosis

After a cardiac injury or disease, cardiac tissue function is reduced due to the activation of cardiac fibroblasts, resulting in cardiac fibrosis [50]. Fibrosis is characterized by the activation and proliferation of fibroblasts and the accumulation of excess extracellular matrix in response to injury or disease processes. Fibroblast activation protein (FAP) is a molecular marker in the hearts of patients with cardiomyopathy [51]. Rurik and colleagues developed FAP-CAR-T cells, which showed the elimination of activated fibroblasts in a mouse model of heart disease, resulting in a significant improvement in cardiac function [52]. In addition, in a mouse model of hypertensive cardiac injury and fibrosis, FAP-CAR-T cells specifically eliminated myofibroblasts, resulting in significantly reduced cardiac fibrosis and restored cardiac function [53]. Currently, CAR-T cell-based therapy for cardiac fibrosis is undergoing animal experimentation, indicating a wide scope for enhancement. Future research could investigate the use of CAR-based therapies for treating cardiac fibrosis, including the innovative potential of CAR-natural killer (CAR-NK) cells and CAR macrophages (CAR-M). These approaches may provide valuable insights and open new therapeutic strategies to address the challenges associated with efficacy and safety limitations in cardiac fibrosis [53].

### 8.3. Infectious Diseases

CAR-T cell therapy presents a promising long-term therapeutic option for patients with chronic viral and opportunistic fungal diseases, although several challenges remain to be addressed. For example, cytotoxic cells redirected against HIV-infected cells using gp120-specific CARs (NCT04648046) or VC-CAR-T cells (NCT03240328) have shown effective cytolysis of latency-reactivated HIV-1-infected CD4+ T cells in individuals under antiretroviral therapy. Epstein-Barr virus (EBV), a highly prevalent human herpesvirus, is another potential target. CAR-T cells directed against EBV-specific antigens, such as LMP1 (Tang) or the gp350 envelope glycoprotein, have demonstrated efficacy in controlling EBV-associated lymphoproliferative disorders [54].

Human cytomegalovirus (HCMV), prevalent in immunocompromised individuals, causes organ-specific diseases like colitis, retinitis, and hepatitis and is linked to immunosuppression, opportunistic infections, and transplant complications such as graft rejection and graft-versus-host disease (GvHD). Promising results have been obtained using CMV CAR-T cells targeting the pentameric complex (gH, gL, UL128, UL130, and UL131A) in vitro and in humanized in vivo models, though further studies are needed to assess their efficacy comprehensively [50,54].

Hepatitis B virus (HBV) also represents a target for CAR-T cell therapy. Preclinical studies using scFv-based CARs against HBsAg demonstrated effective cytolysis of HBV-infected cells in vitro [55]. Similarly, S-protein-specific CAR-T cells successfully controlled viral replication in murine HBV infection models [56].

In fungal infections, CAR-T cells show potential therapeutic utility. For *Aspergillus fumigatus*, CAR-T cells utilizing the extracellular domain of human Dectin-1—a fungal β-glucan receptor—exhibited specificity for laminarin, causing hyphal damage and growth inhibition in vitro and in vivo. CAR-T cells based on monoclonal antibodies targeting *A. fumigatus* hyphae also induced pro-inflammatory cytokine production upon fungal recognition [57].

Lastly, non-viral infections such as *Mycobacterium tuberculosis* may benefit from CAR-T cell therapy. Although no CAR constructs have been developed for this pathogen to date, efforts are underway to identify target antigens suitable for this approach [54].

## 9. Challenges in Identifying Novel Therapeutic Targets for CAR-T Cell Therapy

CAR-T cell therapy has marked a transformative advancement for diseases lacking effective treatments. However, it faces critical challenges, including overcoming immune evasion mechanisms, particularly in solid tumors and autoimmune conditions, and enhancing persistence and functionality to prevent relapses. Manufacturing complexities and high costs further limit accessibility. Additionally, managing toxicities such as cytokine release syndrome (CRS) and neurotoxicity remains essential [30]. Expanding clinical applications requires addressing these hurdles. In discovering new therapeutic targets, one key challenge includes identifying antigens abundantly and consistently expressed on malignant cells while being minimally present in healthy tissues to reduce off-target effects. The heterogeneity of target expression, especially in solid tumors, complicates universal target selection [58]. Immune evasion strategies, such as antigen downregulation and tumor microenvironment suppression, further hinder efficacy [12]. Finally, developing scalable and efficient preclinical models for target validation remains a crucial barrier to advancing CAR-T therapies.

## 10. Advancing CAR-T Cell Therapy: Innovations for Expanding Its Clinical Applications

To reduce toxicity, researchers are exploring the use of CAR-T, which recognizes combinations of antigens instead of a single antigen, which could improve specificity toward cancer cells. For example, some CARs are designed to be activated only in the presence of two specific AML antigens, such as CD33 and CD123, which helps reduce the impact on healthy cells [59,60,61].

Now a days, most CAR-T cells strategies used in clinic studies employ an autologous approach. Although this model allows utilizing the patient’s own cells to avoid rejection and incompatibility issues, it poses significant challenges because manufacturing times are long, the patient’s clinical status may deteriorate during the CAR-T cell production stage, and collecting functional T cells from sick patients who have received toxic treatments previously may fail [62]. Allogeneic CAR-T cells, derived from healthy donors, may provide significant advantages over the autologous approach by (a) off-the-shelf availability, bypassing the manufacturing delays and leukapheresis failures, (b) reducing variability and improving product quality in therapeutic cell products, with a more robust and functionally competent CAR-T cell manufacturing, and (c) the possibility of bulk manufacturing of CAR-T cells, reducing production costs, improving scalability, and making this therapies more accessible [60,62].

Currently, several clinical trials are underway exploring the use of allogeneic CAR-T cell therapies as a strategy to overcome the limitations associated with autologous CAR-T cells (Table 3). These trials aim to address challenges such as manufacturing delays, high production costs, and the variability in product quality observed with patient-derived cells. Allogeneic CAR-T therapies, produced from healthy donors, offer the potential for ‘off-the-shelf’ availability, enabling faster and more uniform treatment delivery. These potential advantages of allogeneic CAR-T cells should be valuated against potential limitations like the high risk of rejection by the host immune system, the development of graft-versus-host-disease, or immune evasion by malignant cells. There is significant research on strategies to avoid these limitations, including gene editing to eliminate endogenous TCRs and knocking out MHC expression in the allogeneic CAR-T cells or the selection and transduction of double-negative T cells from healthy donors. These advancements could significantly expand the accessibility and applicability of CAR-T cell therapy across a broader range of patients and indications [60,61,63,64].

## 11. Discussion

CAR-T cell therapy is a relatively new and cutting-edge treatment that has been classified as a gene therapy product inside the advanced therapy medicinal product (ATMP) according to EMA. Nowadays, there are only six commercialized products that have demonstrated clinical success primarily in treating pediatric and adult hematological malignancies. However, several obstacles have been presented at different levels that still limit its widespread use. Such limitations can be classified, from primary effects of action such as severe toxicities, poor in vivo persistence, antigen escape, heterogeneity, etc., to challenges related to the development of reproducible and consistent production processes as well as economic issues, which, due to the cost, has a high burden nowadays. As described before, every application presents advantages and specific challenges. Addressing these challenges is key to expanding the application of CAR-T cells to a wider range of diseases. Leveraging the potential of the chimeric antigen receptor (CAR) molecular design, various strategies have been developed to overcome these hurdles. The discovery of dual-targeting CAR-T cells, innovations in gene editing techniques, and the search for new targets are all promising strategies to expand the therapy’s application to non-malignant diseases such as autoimmune diseases, cardiac fibrosis, and fungal and viral infections. Thus, the careful selection of target antigens, ensuring they are widely expressed in target cells and minimally expressed in healthy cells, is useful to ensure the therapy’s efficacy and safety. The research progress in identifying and optimizing new targets and customizing CAR-T cells opens new avenues for making CAR-T cell therapy a cornerstone in treating a range of refractory and chronic conditions.

Also, it is important to note that the manufacturing of CAR-T cells as pharmaceutical products continues to face technological, economic, and regulatory challenges in addition to the scientific issues discussed before. Although CAR-T cell therapy has demonstrated clinical efficacy and new approaches indicate its potential to improve the lifespan of patients with refractory or chronic conditions, its development is questionable due to the high production costs, limiting the number of patients who can access its use. Therefore, despite their potential across a wide range of diseases, various areas of opportunity must be addressed to achieve their implementation as a standard treatment in the future. For example, even though CAR-T cells are an individual therapy, the production technology has to be robust and follow the same standards to guarantee the product quality for each patient. In the same way, every production lot has to consider minimal standard parameters for cell expansion. The technology also has to be accessible for patients in non-developed countries, and this is not an easy issue because of the cost of the treatments but also because of the lack of regulation. Developed countries have established regulations that are improvable, and these regulations should be harmonized and adapted to the national needs and requirements of patients in non-developed countries. The cost of the product is also a critical issue. Novel technologies as the use of allogeneic cells is a very feasible option but critical points associated with immune response must be overcome. Along the same line, improvement in product bioprocess and analytical methods to establish the best doses per patient could support the decrease in the final product, considering the different applications of this relatively novel technology.

## 12. Conclusions

Currently, CAR-T cell therapy is one of the most developed advanced therapies for the treatment of hematological diseases, but its potential is spreading to different illnesses, including not only cancer but also chronic as well as infectious diseases. New targets are now in clinical trials and show specific advantages and limitations for each application. While many studies have focused on different targets for cancer, including solid tumors, the search for targets for autoimmune diseases is also increasing, as well as for cardiac and infectious diseases. Although the initial work was with T cells and this is still the main development, it is important to note that different immune cells are now also studied as natural killer cells and macrophages among others, making CAR-T cell therapy a potential tool for improving patient quality; the search for novel therapeutic goals is one aim that will evolve during the next years. However, like any recent therapy, many challenges are present and must be overcome to guarantee access worldwide.

## Figures and Tables

**Table 1:** CAR-T therapies approved by the EMA and FDA until July 2024.

Generic Name	Brand Name	FDA/EMA Approval	Target	Malignancy Indication	Dosage	Cost per Doses (USD)
Tisagenlecleucel	Kymriah	17 August 2017/18 August 2018	CD19	ediatric and young adult patients with B-cell ALL. Adult patients with (r/r) LBCL, DLBCL, HGBL and DLBCL arising from FL.	Patient ≤ 50 kg: 0.2–5 × 10^6^ cells/kg Patient > 50 kg: 0.1–2.5 × 10^8^ cells/kg	475,000
Axicabtagene ciloleucel	Yescarta	18 October 2017/23 August 2018	CD19	Adult patients with (r/r) LBCL, PMBCL, HGBL, and DLBCL arising from FL.	2 × 10^6^/2 × 10^8^ cells/kg	424,000
Brexucabtagene autoleucel	Tecartus	24 July 2020/14 December 2020	CD19	Adult patients with (r/r MCL).	2 × 10^6^/2 × 10^8^ cells/kg	373,000
Idecabtagene vicleucel	ABECMA	26 March 2021/18 August 2021	BCMA	Multiple myeloma.	3–4.6 × 10^8^ cells	419,500
Lisocabtagene maraleucel	Breyanzi	5 February 2021/4 April 2022	CD19	DLBCL, HGBL, PMBCL, and FL grade 3B.	90–110 × 10^6^ cel	487,500
Ciltacabtagene autoleucel	Carvykti	28 February 2022/25 May 2022	BCMA	Multiple myeloma.	0.5–100 × 10^6^ cells/kg	465,000

ALL, Acute lymphocytic leukemia; BCMA, B-Cell maturation antigen; LBCLs, Large B-cell lymphomas; DLBCL, diffuse large B cell lymphoma; HGBL, High-grade B-cell lymphoma; DLBCL, Diffuse large B cell lymphoma; FL, Follicular lymphoma; PMBCL, Primary mediastinal large B-cell lymphoma; MLC, Mantle cell lymphoma.

**Table 2:** Some of the CAR-T therapy novel target antigens that are currently in clinical trials.

Antigen Target	Disease	CAR-T Type	Clinical Trial Identifier
Solid tumors			
ALPP		CAR-T	NCT04627740
FR*α*		CAR-T	NCT03585764
HER2		CAR-T	NCT04511871
MSLN	Ovarian Cancer	CAR-T	NCT03814447, NCT04503980, NCT02580747, NCT03916679, NCT03799913, NCT04562298, NCT03054298 NCT02159716
MUC 16		CAR-T	NCT06469281
TAG72		CAR-T	NCT05225363
GD2	Neuroblastoma	CAR-T	NCT05990751, NCT04637503, NCT03373097, NCT02919046, NCT01822652, NCT03721068, NCT03635632
L1-CAM (CD171)		CAR-T	NCT02311621
B7-H3		CAR-T	NCT05241392, NCT04077866
CD147		CAR-T	NCT04045847
EGFRviii	Glioblastoma	CAR-T E-SYNC	NCT05063682 NCT06186401
IL13R*α*2		CAR-T	NCT04003649
CEA		CAR-T	NCT06010862, NCT04348643, NCT06126406, NCT06006390
EGFR		CAR-T	NCT05341492
GD2		CAR-T	NCT05620342
GPC3		CAR-T	NCT02876978
MSLN	Lung cancer	CAR-T	NCT03054298, NCT04489862
MUC1		CAR-T	NCT03525782
Lewis Y		CAR-T	NCT03851146
PSMA		CAR-T	NCT03198052
ROR1		CAR-T	NCT02706392
CEA		CAR-T	NCT06043466, NCT04348643, NCT05240950
GCC		CAR-T	NCT05319314
GUCY2C	Colorectal cancer	CAR-T	NCT03198052
MSLN		CAR-T	NCT02959151
NK2GD		CAR-T	NCT05248048, NCT04550663
GPC3	Liver cancer	CAR-T	NCT05344664
CEA		CAR-T	NCT06010862, NCT06126406, NCT06006390, NCT04348643
CD133		CAR-T	NCT02541370
B7-H3	Pancreatic cancer	CAR-T	NCT05143151, NCT06158139
CLDN18.2		CAR-T	NCT05620732, NCT05472857
HER2		CAR-T	NCT03740256
MSLN		CAR-T	NCT06054308, NCT01583686
SCA		CAR-T	NCT03267173
CLDN18.2	Gastric cancer	CAR-T	NCT05620732
HER2		CAR-T	NCT04511871
CD44v6		CAR-T	NCT04430595, NCT04427449
c-Met		CAR-T	NCT01837602
EpCAM		CAR-T	NCT02915445
EGFR/B7-H3	Breast cancer	CAR-T	NCT05341492
GD2		CAR-T	NCT04511871 NCT03635632
MUC1		CAR-T	NCT05812326
TRAIL-R2 and HER2		Bi-Specific CAR-T	NCT06251544
CAIX		CAR-T	NCT04969354
CD70	Renal cancer	CAR-T	NCT05420519 NCT06480565
Anti-AXL (CCT301-38)		CAR-T	NCT03393936
Anti-ROR2 (CCT 301-59)		CAR-T	NCT03393936
CD70		CAR-T	NCT02830724
gp100	Melanoma	GPA-TriMAR	NCT03649529
VEGFR-2		CAR-T	NCT01218867
KLK-2		CAR-T	NCT05022849
PSMA	Prostate cancer	CAR-T	NCT04249947, NCT05805371, NCT05354375, NCT06228404,
PSCA		CAR-T	NCT05805371
PSMA/PSCA		CAR-T	NCT03873805 NCT01140373, NCT05732948
Autoimmune diseases			
CD19	Systemic Lupus Erythematosus (SLE)	CAR-T	NCT03030976
CD19	Rheumatoid arthritis	CAR-T	NCT06475495
CD19	SLE, Sjogren’s Syndrome, Systemic Sclerosis, Inflammatory Myopathy, ANCA Associated Systemic Vasculitis, Antiphospholipid Syndrome	U CAR-T	NCT05859997
CD19/CD20	Relapsed and/or Refractory AQP4-IgG Seropositive Neuromyelitis Optica Spectrum Disorders (NMOSD)	tanCAR-T	NCT03605238
	Sjogren’s Syndrome,	CAR-T	NCT05085431
	Scleroderma	CAR-T	NCT05085444
BCMA/CD19	Immune Nephritis	CAR-T	NCT05085418
	SLE	CAR-T	NCT05030779
	Autoimmune Diseases	CAR-T	NCT06428188
BCMA	Generalized Myasthenia Gravis (MG)	CAR-T	NCT04146051
MuSK		CAAR-T	NCT05451212
Dsg3 autoantibodies	Pemphigus Vulgaris	CAAR-T	NCT04422912
Infectious diseases			
gp120	HIV infection	Bi-specific CAR-T	NCT04648046
VRC01		CAR-T	NCT03240328
BRG01	Epstein-Barr Virus (EBV)-Positive Nasopharyngeal Carcinoma	CAR-T	NCT05864924

ALPP, Placental alkaline phosphate; FRα, Folate receptor alpha; HER2, human epidermal growth factor receptor 2; MSLN, mesothelin; MUC, Mucin 16; TAG-72, Tumor associated glycoprotein-72; GD2, glycosphingolipid disialoganglioside; L1-CAM, L1 cell adhesion molecule; B7-H3, B7 homolog 3 protein; CD, cluster of differentiation; EGFR, epidermal growth factor receptor;; IL13Rα2, interleukin 13 receptor subunit alpha 2; GPC-3, Glypican-3; PSMA, prostate-specific membrane antigen; ROR1, Receptor-tyrosine-kinase-like orphan receptor 1; CEA, carcinoembryonic antigen; GUCY2C, intestinal epithelial receptor Guanylyl Cyclase C; GCC, Guanylyl cyclase C; NKG2D, activating immune receptor expressed by NK and effector T cells; CLDN, Claudin; PSCA, Prostate stem-cell antigen; c-MET, C-mesenchymal-epithelial transition factor; EpCAM, epithelial cell adhesion molecule; TRAIL-R2, tumor necrosis factor-related apoptosis-inducing ligand receptor 2; CAIX, Carbonic anhydrase IX; AXL, a receptor tyrosine kinase; ROR2, Receptor tyrosine kinase-like orphan receptor 2; gp100, glycoprotein 100; VEGFR, Vascular Endothelial Growth Factor Receptor; KLK-2, kallikrein-2; BCMA, B cell maturation antigen; MuSK, Muscle-specific tyrosine kinase; gp120, glycoprotein 120; VRC01, anti-HIV antibody; BRG01, n autologous T cell immunotherapy product engineered to express chimeric receptors targeting the Epstein-Barr virus (EBV) antigen on the surface of T cells. CAR-T, Chimeric antigen receptor (CAR) T cells; E-SYNC CAR-T, SynNotch Receptor-Induced Anti-EphA2/IL-13R alpha2 CAR; GPA-TriMAR, a modified chimeric antigen receptor (CAR) that consist of three subunit in its outer membrane domain; tanCAR-T, tandem chimeric antigen receptor (CAR) T cells; UCAR-T, universal chimeric antigen receptor (CAR) T cells; CAAR, chimeric autoantibody receptor.

**Table 3:** Current clinical trials using allogeneic CAR-T cells.

Therapy	Target	Indication	Clinical Trial Identifier	Sponsor
ALLO-501	CD19	Relapsed/Refractory Non-Hodgkin Lymphoma	NCT03939026	Allogene Therapeutics
ALLO-501A	CD19	Relapsed/Refractory Non-Hodgkin Lymphoma	NCT04416984	Allogene Therapeutics
ALLO-715	BCMA	Relapsed/Refractory Multiple Myeloma	NCT04093596	Allogene Therapeutics
UCART19	CD19	Relapsed/Refractory ALL	NCT02746952	Institut de Recherches Internationales Servier
PBCAR0191	CD19	Relapsed/Refractory NHL/ALL	NCT03666000	Imugene Limited
WU-CART-007	CD7	T-cell Malignancies	NCT04984356	Wugen, Inc.
ALLO-647	CD52	Large B-cell lymphoma	NCT05714345	Allogene Therapeutics
Donor-Derived CAR T Cells	CD5	T-Cell Acute Lymphoblastic Leukemia	NCT05032599	Beijing Boren Hospital
MEMCAR19	CD19	Acute Lymphoblastic Leukemia	NCT04881240	St. Jude Children’s Research Hospital
VCAR33	CD33	Acute Myeloid Leukemia	NCT05984199	Vor Biopharma

CD, cluster of differentiation; BCMA, B-Cell Maturation Antigen.
